# Machine learning models for dementia screening to classify brain amyloid positivity on positron emission tomography using blood markers and demographic characteristics: a retrospective observational study

**DOI:** 10.1186/s13195-024-01650-1

**Published:** 2025-01-21

**Authors:** Noriyuki Kimura, Kotaro Sasaki, Teruaki Masuda, Takuya Ataka, Mariko Matsumoto, Mika Kitamura, Yosuke Nakamura, Etsuro Matsubara

**Affiliations:** 1 Department of Neurology, Faculty of Medicine, Oita University, 1-1 Idaigaoka, Hasama-machi, Yufu, Oita, 879-5593 Japan; 2 Human Biology Integration Foundation, Deep Human Biology Learning, Eisai Co., Ltd, 4-6-10 Koishikawa, Bunkyo-ku, Tokyo, 112-8088 Japan; 3 Neurology Department, Medical Headquarters, Eisai Co., Ltd, 3-7-1 Nishi Shinjuku, Shinjuku-ku, Tokyo, 163-1023 Japan

**Keywords:** AD, Alzheimer’s disease, Amyloid β positivity, Dementia, Machine learning

## Abstract

**Background:**

Intracerebral amyloid β (Aβ) accumulation is considered the initial observable event in the pathological process of Alzheimer’s disease (AD). Efficient screening for amyloid pathology is critical for identifying patients for early treatment. This study developed machine learning models to classify positron emission tomography (PET) Aβ-positivity in participants with preclinical and prodromal AD using data accessible to primary care physicians.

**Methods:**

This retrospective observational study assessed the classification performance of combinations of demographic characteristics, routine blood test results, and cognitive test scores to classify PET Aβ-positivity using machine learning. Participants with mild cognitive impairment (MCI) or normal cognitive function who visited Oita University Hospital or had participated in the USUKI study and met the study eligibility criteria were included. The primary endpoint was assessment of the classification performance of the presence or absence of intracerebral Aβ accumulation using five machine learning models (i.e., five combinations of variables), each constructed with three classification algorithms, resulting in a total of 15 patterns. L2-regularized logistic regression, and kernel Support Vector Machine (SVM) and Elastic Net algorithms were used to construct the classification models using 34 pre-selected variables (12 demographic characteristics, 11 blood test results, 11 cognitive test results).

**Results:**

Data from 262 records (260 unique participants) were analyzed. The mean (standard deviation [SD]) participant age was 73.8 (7.8) years. Using L2-regularized logistic regression, the mean receiver operating characteristic (ROC) area under the curve (AUC) (SD) in Model 0 (basic demographic characteristics) was 0.67 (0.01). Classification performance was similar in Model 1 (basic demographic characteristics and Mini Mental State Examination [MMSE] subscores) and Model 2 (demographic characteristics and blood test results) with a cross-validated mean ROC AUC (SD) of 0.70 (0.01) for both. Model 3 (demographic characteristics, blood test results, MMSE subscores) and Model 4 (Model 3 and ApoE4 phenotype) showed improved performance with a mean ROC AUC (SD) of 0.73 (0.01) and 0.76 (0.01), respectively. In models using blood test results, thyroid-stimulating hormone and mean corpuscular volume tended to be the largest contributors to classification. Classification performances were similar using the SVM and Elastic Net algorithms.

**Conclusions:**

The machine learning models used in this study were useful for classifying PET Aβ-positivity using data from routine physician visits.

**Trial registration:**

UMIN Clinical Trials Registry (UMIN000051776, registered on 31/08/2023).

**Supplementary Information:**

The online version contains supplementary material available at 10.1186/s13195-024-01650-1.

## Background

Dementia is a growing public health concern given increases in life expectancy and population aging, and has serious social and economic effects on both patients and their caregivers [1]. In people over 65 years of age, Alzheimer’s disease (AD) is a major cause of dementia [1]. The accumulation of amyloid β (Aβ) in the brain, a key characteristic of AD, is widely considered the initial observable event in the AD pathological process [2]. Other neuropathologic hallmarks of AD include neurofibrillary tangles and neuronal degeneration [2]. Biomarker research has focused on tracking the progression of AD through the accumulation of Aβ and tau in the brain, which are associated with neurodegeneration and cognitive decline [3, 4].

Recently, three anti-Aβ antibodies, aducanumab, lecanemab, and donanemab, were approved by the Food and Drug Administration for the treatment of patients with mild cognitive impairment (MCI) or mild dementia due to AD (note that aducanumab is not currently on the market) [5–7]. According to the package inserts [7–9], confirmation of the presence of Aβ pathology is required prior to treatment initiation. Therefore, accurate detection of amyloid pathology in individuals with MCI is essential to maximize the benefit of these anti-Aβ antibody therapies in future clinical practice [10].

^11^C-Pittsburgh Compound-B (PiB) positron emission tomography (PET) and measurement of cerebrospinal fluid (CSF) Aβ levels are established amyloid biomarkers that can predict the incidence of AD before the onset of dementia [11]. Aβ was first isolated and quantified from CSF in 1992 [12]. In 2004, Klunk et al. first demonstrated PiB-PET (amyloid PET), which has been widely used in AD research for over 20 years [13]. However, both techniques are unsuitable as screening tools in clinical settings owing to the high cost, radiation exposure, and the need for invasive lumbar puncture. Considering the low prevalence of Aβ-positive MCI, which has been shown to range from 22.0% in a population-based study to 46.6% in a multicenter research study, performing PET screening on all patients with suspected AD may translate to unnecessary increased costs and patient screening burden [14, 15].

To improve early identification of patients who may benefit from AD treatments, it is important to develop a simple and efficient method for screening that uses universally available data such as demographic characteristics, routinely collected blood data, and cognitive scores [16, 17]. Blood-based biomarkers (e.g., p-tau217, p-tau181, or Aβ42/40) have shown promise in their ability to predict Aβ pathology [18–21]; however, advanced technologies such as mass spectrometry and immunoassays are needed to achieve highly precise and reproducible plasma Aβ measurements [20, 22, 23]. Previous studies have shown a possible association between the presence of Aβ and variables measured in routine blood testing [24–43]. Additionally, age is one of the most important risk factors for developing AD [44]. The development of a predictive model to detect amyloid pathology using blood data collected as part of routine medical practice would provide an important new tool that could be implemented worldwide.

In recent years, there has been a focus on using machine learning to build models that are able to predict PET Aβ-positivity, MCI, and risk of future PET Aβ-positivity [10, 45–48]. Although some progress has been made in developing predictive models for Aβ pathology and early detection of AD, studies using a combination of data collected in regular clinical practice (e.g., demographic characteristics, lifestyle factors, and blood test results) to make predictions/classifications are limited. In the present study, we used data obtainable from medical health practice and physician visits, including blood tests and cognitive test scores, to detect individuals with elevated intracerebral Aβ accumulation. To the best of our knowledge, few studies have focused on routine blood data as variables for identifying individuals at high risk for intracerebral Aβ accumulation.

This study aimed to develop machine learning models to classify PET Aβ-positivity in patients with preclinical AD, defined as a state of clinically normal cognitive function with the presence of pathologic changes of AD [49], and prodromal AD from patient demographic characteristics and routinely collected blood data accessible to family physicians.

## Methods

### Study design

This was a retrospective observational study to assess the classification performance of combinations of variables (demographic characteristics, blood test results, and cognitive test scores) that may be useful for screening Aβ accumulation in the brains of people with MCI or normal cognitive function using machine learning. Prior to initiation, the study protocol was approved by the local ethics committee of Oita University Hospital (approval number 2574; approval date, June 22, 2023). This study used existing patient information, and it was difficult to obtain informed consent. Thus, an information disclosure document that included information on the conduct and purpose of the study was made publicly available on the university website [50]. This document was approved by the ethics committee and authorized by the head of Oita University Hospital. The study was registered with the University hospital Medical Information Network Clinical Trials Registry (UMIN000051776 on August 31, 2023).

### Participants

This study used two data sources. One data source was patients who visited Oita University Hospital (September 1, 2012, to November 30, 2017, [5 years, 3 months]), and the other was elderly residents in Usuki City, Oita Prefecture, Japan, who participated in the USUKI study (USUKI study cohort; October 1, 2015, to November 30, 2017, [2 years, 2 months]). Participants with MCI or normal cognitive function and who met the inclusion/exclusion criteria of the present study were eligible for this analysis. The USUKI study was a prospective study of community-dwelling older adults without dementia designed to explore the risk and protective lifestyle factors of cognitive decline later in life. The study design and details of the methods have been published previously [51, 52]. The inclusion criteria for the present study were (1) a clinical dementia rating (CDR) of 0 or 0.5; and (2) availability of Aβ PiB-PET data. Individuals who met the following criteria were excluded: (1) insufficient data for analysis, (2) an indication of refusal to participate in the study, or (3) deemed ineligible for participation by the principal investigator or subinvestigators (e.g., data with many missing or abnormal values or data for suspected dementia). For criterion 3), data were reviewed prior to analysis, and depending on the situation, participants with a large number of missing or abnormal values were excluded. Abnormal values were those that, as judged from a clinical perspective by an expert, could not be obtained because of measurement errors or other factors. Blood test results that exceeded the standard value were not considered abnormal if they were clinically relevant. Among people who met the eligibility criteria, those whose available data satisfied the following were included in the analysis set: (1) all measurements for the variables included in the classification analysis (demographic characteristics, blood tests, MMSE, and CDR) were collected ≤ 365 days from the outcome measurement (PiB-PET scan) date, and (2) no missing values in the study blood test results.

### Study endpoint

The primary endpoint was the classification performance of the presence or absence of intracerebral Aβ accumulation using five different machine learning models. The true label for positive Aβ accumulation, or the positive presence of intracerebral Aβ accumulation, was defined as a mean PiB standardized uptake value ratio (SUVR) of ≥ 1.2.

### Model construction and evaluation

The methods used for model construction were similar to those previously described by Kimura et al. [10]. Positive and negative Aβ accumulation in the brain was used as the objective variable. The classification model was developed using data that had been imputed for missing values. Missing values for continuous variables were imputed using the median value, which was calculated for the entire dataset. Missing values for categorical variables were imputed using the mode value (i.e., the value that appeared most frequently in the dataset). As a sensitivity analysis to the missing value completion method, the model was also built and evaluated using a dataset in which missing value completion was performed using the K-nearest neighbors (KNN) method (number of neighbors was set at 5). The computation of these completion values was performed using the entire dataset (i.e., calculated for the entire dataset). Three algorithms previously found by Kimura et al. to be effective in predicting Aβ accumulation were selected as the basis for constructing the models: L2-regularized logistic regression, kernel Support Vector Machine (SVM), and Elastic Net [10]. The radial basis function kernel was used as the kernel function in the SVM algorithm in the present study. Thirty-four variables were pre-selected for use in the classification modeling based on existing literature [24–43], which included 12 demographic characteristics, 11 blood test results, and 11 cognitive test results. The 12 demographic characteristics comprised age, sex, years of education, body mass index (BMI), current alcohol consumption, smoking status (past or present), and six medical history variables (hypertension, hyperlipidemia, heart disease [e.g., heart failure, arrhythmia], stroke, diabetes mellitus, and thyroid disease). The 11 blood test results were apolipoprotein E4 (ApoE4) phenotyping (E4+/−), liver function markers (aspartate aminotransferase [AST] to alanine transaminase [ALT] ratio, alkaline phosphatase [ALP], gamma-glutamyl transferase [γ-GTP], total bilirubin), renal function markers (estimated glomerular filtration rate [eGFR]), thyroid-stimulating hormone (TSH), white blood cell (WBC) count, hemoglobin concentration, mean corpuscular volume (MCV), and platelet count. The 10 markers other than ApoE4 phenotyping were typically measured during routine physician visits. The cognitive test results included 11 Mini Mental State Examination (MMSE) subscores. Note that in this study, the MMSE total score was not used for classification, but rather the subscores were explored for their individual classification value.

For these pre-selected variables, continuous variables were transformed into quantile scales [53], and categorical variables were used as dummy variables using one-hot encoding. The conversion to quantile scales was used because it was previously found to be an effective method for improving prediction accuracy [10]. To avoid the overestimation of classification performance, the machine learning models were trained and validated using a 5-fold cross-validation scheme. For each round of validation, participants were randomly divided into five groups. Four groups of data were used as learning sets to train the models, and the classification performance of the trained model was evaluated using the remaining data set (validation set). This process was repeated for each of the groups (i.e., data from each of the five groups were used as the validation set). To reduce bias in the data split, 15 different split data values were created based on 15 seed values for which the 5-fold cross-validation process was performed. The analysis outputs classification performance results for five folds for each of the 15 seeds, totaling 75 values, and these values are averaged for each seed to obtain 15 cross-validated mean values. This method was applied across five models and three classification algorithms. Finally, the mean (standard deviation [SD]) of the classification performance output values (15 cross-validated mean values) was calculated. Classification performance was evaluated using receiver operating characteristic (ROC) area under the curve (AUC) values, sensitivity, specificity, positive predictive value, negative predictive value, and accuracy. The Youden index calculated from learning sets was used to determine the cut-off point for calculating these evaluation indices in 5-fold cross-validation. The Boruta method was used as the variable selection technique to eliminate variables that did not contribute to the classification performance of the model [54]. In the Boruta method, the contribution of each variable was calculated as permutation importance [55]. Variables were selected in the training models without the use of the validation set to avoid overfitting and biased evaluation of classification performance. Hyper parameter tuning was conducted by grid search for each learning set, in which Log-Loss was used as the objective function.

Model 0 used four participant demographic characteristic variables: age, sex, years of education, and BMI. These were the most easily obtained variables and were used as benchmarks in Model 0 to evaluate classification performance. Note that the Boruta method was not used in Model 0 because it included only four variables. Model 1 variables were those included in Model 0 plus MMSE subscores. Model 2 variables were those included in Model 0 plus blood test results (excluding ApoE4 phenotype) and the other demographic characteristics (medical history, current alcohol consumption, and smoking status). Model 3 variables were those included in Model 2 plus MMSE subscores. Model 4 variables were those included in Model 3 plus ApoE4 phenotype. ApoE4 phenotype is not measured in routine clinical practice; therefore, we note that classifications by Model 4 cannot be made using routine medical data alone. Using the classification models generated as described above, a diagram of the variable importance calculated based on Shapley Additive exPlanations (SHAP) and a SHAP summary plot [56, 57] were developed. LinearExplainer was used to calculate SHAP values. The variable importance was calculated as an average of the absolute SHAP values.

### Statistical analysis

Considering study feasibility, the number of cases was set so that as many patients as possible could be enrolled. The target number of cases was set at 200, and the maximum number of cases was pre-planned to be 283.

The analysis of the primary endpoint was performed on the analysis set (262 records representing 260 unique participants). To assess the distribution balance of participant background characteristics between the Aβ positive and negative groups, Welch’s two-sample two-sided t-test was used for continuous variables and the chi-square test was used for categorical variables. R version 4.3.1 with the tableone package 0.13.2 (The R Foundation for Statistical Computing, Vienna, Austria) was used to calculate descriptive statistics for participant background characteristics. Python version 3.7.12 with the scikit-learn 1.0.2 and shap 0.41.0 packages (Python Software Foundation, Wilmington, DE, USA) was used for model construction and evaluation.

## Results

### Participants

**Fig. 1:**
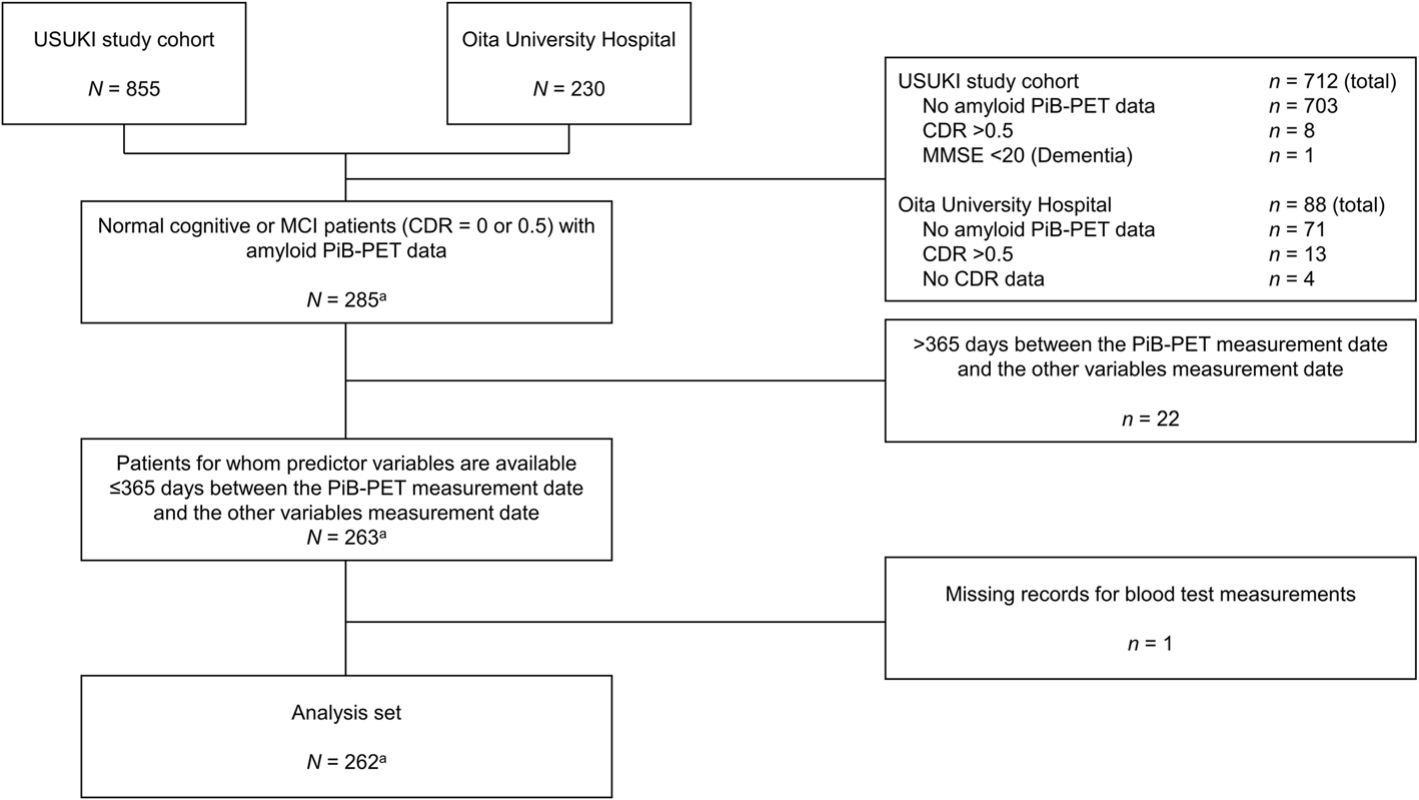
Participant disposition ^a^262 records from a total of 260 unique participants *CDR* clinical dementia rating, *MCI* mild cognitive impairment, *PiB-PET*^11^C-Pittsburgh Compound-B positron emission tomography

Of the 855 patients in the USUKI cohort and the 230 patients who visited Oita University Hospital, 703 and 71, respectively, were excluded because they did not have amyloid PiB-PET data, and 8 and 13, respectively, because they had a CDR > 0.5; additionally, 1 case in the USUKI cohort was excluded because of an MMSE score < 20, indicating dementia, and 4 in Oita University Hospital because they did not have CDR data. No participants were excluded because of a large number of missing or abnormal values. The participant with the most missing values had 11 of 34 variables missing (11 MMSE subscores). Prior to analysis, it was determined that exclusion of this participant because of the high number of missing values was not necessary. Note that out of 285, two participants overlapped across the two cohorts, and therefore the dataset screened for eligibility included 283 unique participants. The overlapping records for the two participants were treated as separate records because the PiB-PET scan dates were approximately 4 years apart between the two cohorts. Overall, 285 records of participants with MCI or normal cognitive function were screened for eligibility and 262 (260 unique participants) were included in the analysis set; 22 were excluded because there was a > 365-day period between their PiB-PET and the collection of data related to other variables, and one patient in the USUKI cohort was excluded because of missing blood test records (Fig. 1).

Participant background characteristics before imputing missing data are shown in Table 1. In total, 38.5% (101/262) of participant records were Aβ positive, 79.8% (209/262) had MCI (CDR = 0.5), and 20.2% (53/262) were cognitively normal (CDR = 0). The mean (SD) age was 73.8 (7.8) years, 51.9% (136/262) were male, and the mean (SD) MMSE score was 26.3 (2.4). Blood test results showed that 19.5% (50/262) of participant records were ApoE4 positive. Values for the AST to ALT ratio, total bilirubin, TSH, hemoglobin, and MCV were significantly different between the two groups (*p* < 0.05). Background characteristics after imputing missing data are shown in Table S1 (Additional file 1). There were a total of 12 records with missing values for any of the variables.

**Table 1:** Background characteristics of study participants

	Total *N* = 262^a^	Missing *n* (%)	Amyloid β positive *n* = 101	Amyloid β negative *n* = 161	SMD	*p*-value^b^
**Demographic, lifestyle, and clinical characteristics**						
Age, years, mean (SD)	73.8 (7.8)	0 (0)	76.2 (5.6)	72.3 (8.5)	0.54	< 0.001
Sex, *n* (%)						
Male	136 (51.9)	0 (0)	62 (61.4)	74 (46.0)	0.31	0.021
Years of education, mean (SD)	11.8 (2.2)	0 (0)	11.7 (1.9)	11.8 (2.4)	0.08	0.520
BMI, kg/m^2^, mean (SD)	22.81 (3.07)	0 (0)	22.27 (3.07)	23.15 (3.03)	0.29	0.024
Current alcohol consumption, *n* (%)	94 (36.4)	4 (1.5)	31 (31.3)	63 (39.6)	0.17	0.224
Smoking status^c^, *n* (%)	97 (37.6)	4 (1.5)	29 (29.3)	68 (42.8)	0.28	0.041
Medical history, *n* (%)						
Hypertension	136 (51.9)	0 (0)	53 (52.5)	83 (51.6)	0.02	0.985
Hyperlipidemia	89 (34.0)	0 (0)	42 (41.6)	47 (29.2)	0.26	0.054
Heart disease	36 (13.7)	0 (0)	19 (18.8)	17 (10.6)	0.23	0.088
Stroke	18 (6.9)	0 (0)	8 (7.9)	10 (6.2)	0.07	0.778
Diabetes mellitus	42 (16.0)	0 (0)	9 (8.9)	33 (20.5)	0.33	0.021
Thyroid disease	21 (8.0)	0 (0)	12 (11.9)	9 (5.6)	0.22	0.111
**Blood test results**						
ApoE4 positive, *n* (%)	50 (19.5)	6 (2.3)	34 (35.1)	16 (10.1)	0.63	< 0.001
AST to ALT ratio, mean (SD)	1.37 (0.46)	0 (0)	1.43 (0.38)	1.34 (0.50)	0.22	0.081
ALP, U/L, mean (SD)	237.2 (72.2)	0 (0)	235.7 (71.8)	238.1 (72.6)	0.03	0.795
γ-GTP, U/L, mean (SD)	27.11 (21.85)	0 (0)	26.44 (22.54)	27.54 (21.47)	0.05	0.695
Total bilirubin, mg/dL, mean (SD)	0.661 (0.281)	0 (0)	0.608 (0.198)	0.694 (0.319)	0.32	0.008
eGFR, mL/min/1.73m^2^, mean (SD)	66.33 (15.35)	0 (0)	64.29 (16.75)	67.61 (14.31)	0.21	0.100
TSH, µIU/mL, mean (SD)	2.442 (1.938)	0 (0)	2.147 (1.348)	2.627 (2.214)	0.26	0.030
WBC, 1 × 10^3^/µL, mean (SD)	5.604 (1.420)	0 (0)	5.432 (1.244)	5.711 (1.513)	0.20	0.106
Hemoglobin, g/dL, mean (SD)	13.53 (1.60)	0 (0)	13.19 (1.35)	13.75 (1.71)	0.37	0.003
MCV, fL, mean (SD)	94.51 (5.51)	0 (0)	95.69 (6.12)	93.77 (4.96)	0.34	0.009
Platelets, 1 × 10^3^/µL, mean (SD)	209.8 (53.2)	0 (0)	213.3 (57.6)	207.7 (50.4)	0.10	0.420
**Cognitive test scores**						
MMSE, mean (SD)	26.3 (2.4)	0 (0)	25.7 (2.5)	26.7 (2.3)	0.43	0.001
Orientation time	4.5 (0.8)	6 (2.3)	4.3 (1.0)	4.7 (0.6)	0.40	0.003
Orientation place	4.7 (0.6)	6 (2.3)	4.5 (0.7)	4.8 (0.5)	0.47	0.001
Registration	3.0 (0.1)	6 (2.3)	3.0 (0.1)	3.0 (0.1)	0.02	0.856
Attention	3.4 (1.6)	6 (2.3)	3.5 (1.5)	3.4 (1.6)	0.09	0.497
Delayed recall	2.0 (1.0)	6 (2.3)	1.7 (1.0)	2.2 (1.0)	0.46	< 0.001
Language naming	2.0 (0.0)	6 (2.3)	2.0 (0.0)	2.0 (0.0)	< 0.01	-^d^
Language repetition	0.9 (0.2)	6 (2.3)	0.9 (0.3)	1.0 (0.2)	0.12	0.381
Language command	2.9 (0.3)	6 (2.3)	2.9 (0.4)	2.9 (0.3)	< 0.01	0.986
Language read and obey	1.0 (0.1)	6 (2.3)	1.0 (0.1)	1.0 (0.1)	0.07	0.557
Language writing	0.9 (0.3)	6 (2.3)	0.9 (0.3)	0.9 (0.3)	0.06	0.620
Copy	0.9 (0.2)	6 (2.3)	0.9 (0.3)	1.0 (0.2)	0.15	0.271
CDR, *n* (%)						
0 (NC)	53 (20.2)	0 (0)	11 (10.9)	42 (26.1)	0.40	0.005
0.5 (MCI)	209 (79.8)	0 (0)	90 (89.1)	119 (73.9)	0.40	0.005
**Outcome**						
SUVR on PiB, mean (SD)	1.23 (0.53)	0 (0)	1.81 (0.42)	0.87 (0.09)	3.06	< 0.001

^a^262 records from a total of 260 unique participants

^b^Welch’s two-sample two-sided t-test for continuous variables; chi-square test for categorical variables

^c^Past or present

^d^Data were not available

*ALP* alkaline phosphatase, *ALT* alanine transaminase, *ApoE4* apolipoprotein E4, *AST* aspartate aminotransferase, *BMI* body mass index, *CDR* clinical dementia rating, *eGFR* estimated glomerular filtration rate, *MCI* mild cognitive impairment, *MCV* mean corpuscular volume, *MMSE* Mini Mental State Examination, *NC* normal cognitive function, *PiB*^11^C-Pittsburgh Compound-B, *SD* standard deviation, *SMD* standardized mean difference, *SUVR* standardized uptake value ratio, *TSH* thyroid-stimulating hormone, *WBC* white blood cell, *γ-GTP* gamma-glutamyl transferase

### Outcomes

**Fig. 2:**
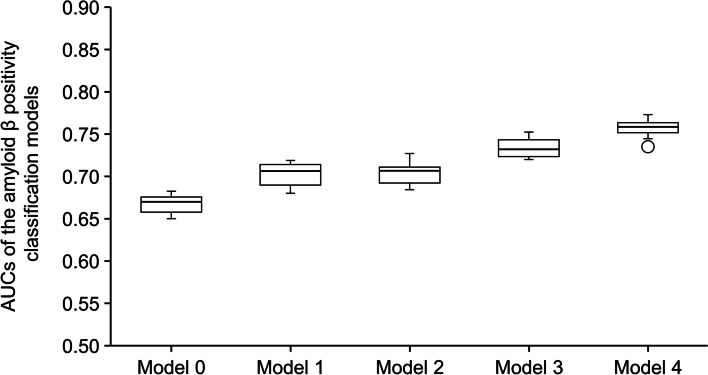
Cross-validated area under the curves of amyloid β positivity classification models: L2-regularized logistic regression Model 0: demographic characteristics (age, sex, body mass index, years of education) Model 1: Model 0 plus all MMSE subscores Model 2: Model 0 plus blood test results (excluding ApoE4 phenotype) and the other demographic characteristics (medical history, current alcohol consumption, and smoking status) Model 3: Model 2 plus all MMSE subscores Model 4: Model 3 plus ApoE4 phenotype Horizontal lines are median values, upper and lower box edges show the first quartile (Q1) and the third quartile (Q3), and upper and lower whiskers represent the 1st quartile − 1.5 × the IQR and 3rd quartile + 1.5 × the IQR, respectively. Circle point is an outlier beyond the whisker range *ApoE4* apolipoprotein E4, *AUC* area under the curve, *IQR* interquartile range, MM*SE* Mini Mental State Examination

**Fig. 3:**
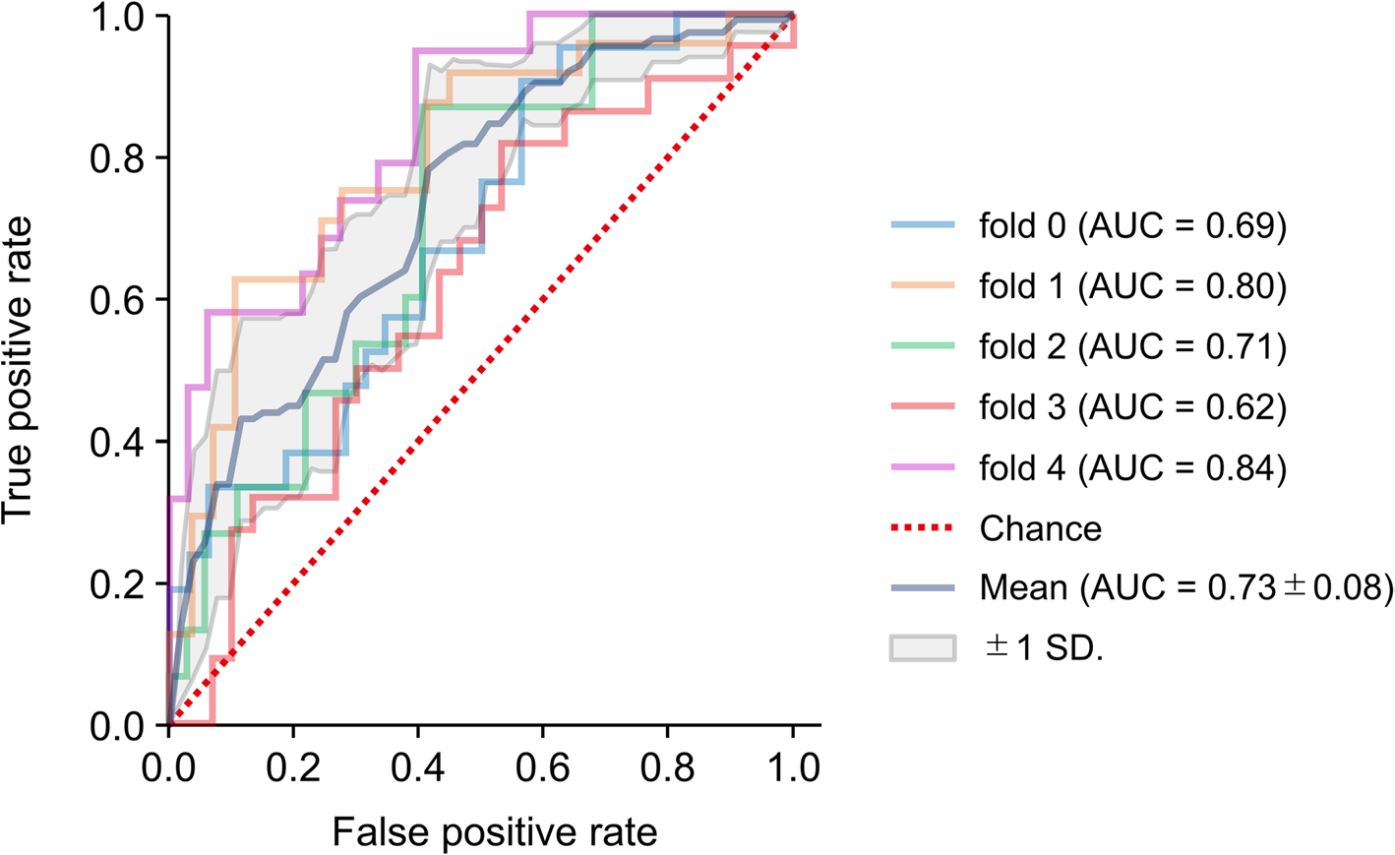
Receiver operating characteristic curve of amyloid β positivity classification Model 3: L2-regularized logistic regression *AUC* area under the curve, *SD* standard deviation

Classification performance findings using the L2-regularized logistic regression are shown in Table 2; Fig. 2. The mean (SD) ROC AUC for Model 0, which used the demographic characteristics of age, sex, BMI, and years of education, was 0.67 (0.01). Performance improved with the use of the 11 MMSE subscores plus the four basic variables (age, sex, BMI, and years of education) of demographic characteristics (Model 1; mean [SD] ROC AUC, 0.70 [0.01]). Performance similarly improved when the demographic characteristics and blood test results (except for ApoE4 phenotype) were used (Model 2; mean [SD] ROC AUC, 0.70 [0.01]). An increase in performance was found when the 11 MMSE subscores were combined with the blood test results (except for ApoE4 phenotype) and demographic characteristics (Model 3; mean [SD] ROC AUC, 0.73 [0.01]) (Fig. 3). Finally, the best performance was observed when ApoE4 phenotype was added to the variables used in Model 3 (Model 4; mean [SD] ROC AUC, 0.76 [0.01]).

**Table 2:** Performance of the amyloid β positivity classification models using L2-regularized logistic regression

	ROC AUC	Sensitivity	Specificity	PPV	NPV	Accuracy
**Model 0**^**a**^	0.67 (0.01)	0.64 (0.04)	0.62 (0.04)	0.52 (0.02)	0.74 (0.01)	0.63 (0.02)
**Model 1**^**b**^	0.70 (0.01)	0.68 (0.04)	0.60 (0.04)	0.52 (0.02)	0.75 (0.02)	0.63 (0.01)
**Model 2**^**c**^	0.70 (0.01)	0.60 (0.04)	0.68 (0.05)	0.56 (0.04)	0.74 (0.01)	0.66 (0.03)
**Model 3**^**d**^	0.73 (0.01)	0.62 (0.04)	0.69 (0.04)	0.57 (0.03)	0.75 (0.01)	0.66 (0.02)
**Model 4**^**e**^	0.76 (0.01)	0.64 (0.03)	0.75 (0.03)	0.62 (0.03)	0.77 (0.01)	0.70 (0.02)

Data are mean (standard deviation)

^a^Model 0: demographic characteristics (age, sex, body mass index, years of education)

^b^Model 1: Model 0 plus all MMSE subscores

^c^Model 2: Model 0 plus blood test results (excluding ApoE4 phenotype) and the other demographic characteristics (medical history, current alcohol consumption, smoking status)

^d^Model 3: Model 2 plus all MMSE subscores

^e^Model 4: Model 3 plus ApoE4 phenotype

*ApoE4* apolipoprotein E4, *MMSE* Mini Mental State Examination, *NPV* negative predictive value, *PPV* positive predictive value, *ROC AUC* receiver operating characteristic area under the curve

**Fig. 4:**
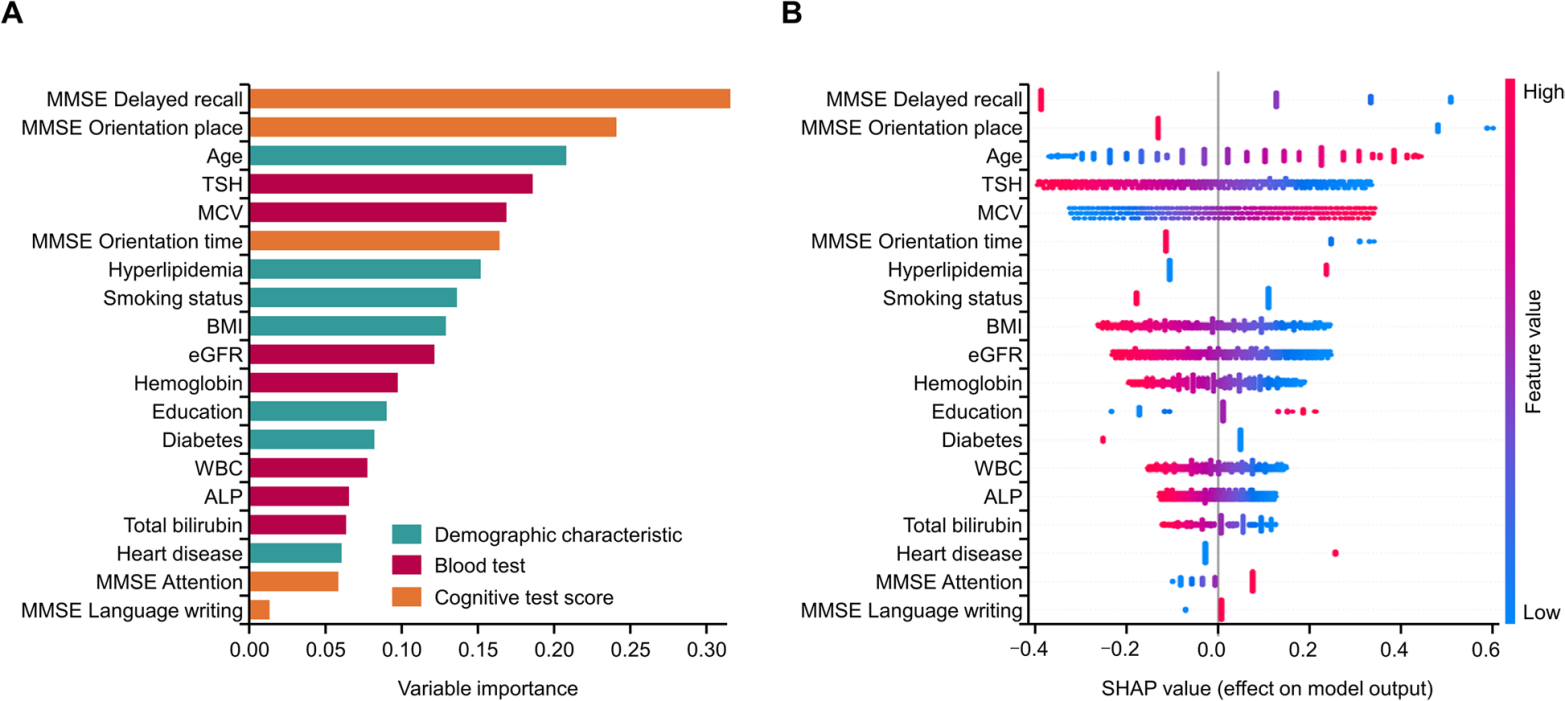
Variable importance (**A**) and Shapley Additive exPlanations summary plot (**B**): L2-regularized logistic regression Model 3 *ALP* alkaline phosphatase, *BMI* body mass index, *eGFR* estimated glomerular filtration rate, *MCV* mean corpuscular volume, *MMSE* Mini Mental State Examination, *SHAP* Shapley Additive exPlanations, *TSH* thyroid-stimulating hormone, *WBC* white blood cell count

The classification performance results when using the kernel SVM and Elastic Net algorithms were not different from those reported for the L2-regularized logistic regression algorithm (Table S2 in Additional file 1). The Elastic Net results mirrored those using the L2-regularized logistic regression, while the ROC AUC values using the kernel SVM tended to be lower, with the exception of those reported for Model 2 (mean [SD] ROC AUC, 0.72 [0.01]). The results when missing values were imputed by KNN were not notably different from the results when missing values were imputed by median value or mode value (Table S3 in Additional file 1). Figure 4 shows the variable importance and SHAP summary plot results for Model 3 trained by the L2-regularized logistic regression algorithm. MMSE subscores for delayed recall and orientation place, along with age, TSH, and MCV, were the five most important variables (Fig. 4A). The SHAP summary plot shows the direction and magnitude of the contribution of each variable to the classification (Fig. 4B). As an example, TSH contributes to the model in that the higher the value, the lower the positive probability, whereas with MCV, the higher the value, the higher the positive probability. Variable importance for Models 0–2 and 4 using the L2-regularized logistic regression algorithm is shown in Figure S1 (Additional file 1).

Considering the blood test variables, TSH and MCV tended to be the largest contributors to the classification across Models 2, 3, and 4. Trends for TSH and MCV were consistent across the models, which was not the case for the other variables. Hemoglobin, ALP, total bilirubin, AST to ALT ratio, eGFR, and WBC were selected and made moderate contributions to some models. Some demographic characteristic variables consistently showed large contributions, including age, BMI, and years of education in Models 2–4. The medical history variables of hyperlipidemia, diabetes, and heart disease were consistent contributors. Delayed recall, orientation place, and orientation time were consistently ranked as the top three contributors (in that order) for MMSE subscores (Models 1, 3, and 4). Language writing, language repetition, attention, and copy were also noted in Models 1, 3, and 4 but had a lower contribution than the top three contributors for MMSE subscores.

## Discussion

This is the first study to develop a model using machine learning techniques to classify amyloid PET positivity using only the results of blood tests routinely performed in dementia diagnosis. Our results provide several novel insights into the social implementation of blood biomarkers for classifying amyloid PET positivity. First, the AUC of the machine learning model using demographic and general blood test data for classifying PET Aβ-positivity was 0.70. Second, the classification models included several variables associated with anemia, vascular risk factors, thyroid hormone, and liver and kidney function. Our results suggest that machine learning models using demographic and general blood test data can supplement amyloid PET and CSF analysis.

Many studies have reported on plasma biomarkers for AD, which can be measured with high accuracy and reproducibility using advanced technologies such as liquid chromatography mass spectrometry and immunoassay methods such as Single Molecule Array (SIMOA) assays. Such plasma biomarkers may include metabolic changes, neurofilament light, p-tau, glial fibrillary acidic protein, signaling proteins related to hematopoiesis, immune response, apoptosis, and neuroprotection, and others [58–66]. Although blood-based AD biomarkers have great potential in the diagnostic work-up of AD, they are not currently useful in routine clinical practice. Despite the numerous studies on various AD-related blood biomarkers in recent years, few have evaluated the classification value of a combination of participant demographic, lifestyle, and general blood test data in routine medical practice for classifying PET Aβ-positivity.

The model developed in the present study (Model 2) was able to classify the AD pathology of amyloid accumulation in the brain using data obtained during routine dementia care, including participant demographic and general blood test data. Model 2 had a classification performance of AUC = 0.70, which was comparable to the performance of Model 1 utilizing MMSE subscores (AUC = 0.70). Therefore, screening for Aβ-positive PET can be achieved using blood test data measured in routine clinical practice. Amyloid PET and CSF testing are expensive and highly invasive, thus limiting their usefulness in clinical practice. In search of more clinically useful options, previous studies have developed amyloid-positive prediction/classification models using cost-effective variables (e.g., demographic characteristics, ApoE genotype, imaging tests, and blood biomarkers) and machine learning that have achieved AUC values > 0.70. However, these models incorporate markers and other variables that are not measured in usual clinical practice, such as ApoE genotype, imaging data, chemokine ligand 13, pancreatic polypeptide, and vascular cell and adhesion protein-1 [18, 67–70]. We note that the AUC of these previously reported models is higher than that of Model 2 in the present study. However, our model, which uses combinations of readily available data, should be a suitable and successful screening tool for amyloid positivity in the primary care setting. When comparing Model 4 with previously reported models using ApoE4 data as a predictive variable, Model 4 had an AUC of 0.76, which demonstrated better performance than some (Petersen et al. AUC, 0.73 [69] and Kim et al. AUC, 0.71 [70]), but not all previously reported models (Palmqvist et al. AUC, 0.80–0.82 [67]). No studies were identified that developed models with substantially better performance than Model 2, which was constructed using only items measured in routine practice. It is important to note that plasma Aβ can be more accurately and reproducibly measured using mass spectrometry and immunoassays [22] than using a machine learning model. One review reported that, among studies using an immunoprecipitation-mass spectrometry assay of Aβ42/Aβ40 with a PET reference, the weighted average of AUC values across 21 cohorts was 0.834 [71]. However, there are limitations in the diagnostic value of the Aβ42/Aβ40 ratio without PET imaging due to overlap between Aβ-positive and -negative patients [72, 73]. Our machine learning model may be useful for addressing this, as it could be used for population-based screening to detect Aβ-positive individuals, which would reduce testing costs and the number of patients who undergo unnecessary amyloid PET imaging.

Our classification models included several variables associated with anemia (MCV and hemoglobin), vascular risk factors (lipid abnormalities, smoking, diabetes, BMI, and ischemic heart disease), and measures of thyroid (TSH), liver (ALP and bilirubin), and kidney (eGFR) function, all of which are associated with Aβ accumulation. In particular, variables used in our models that were shown to be important using L2-regularized logistic regression analysis include age, years of education, vascular risk factors, heart disease, liver and renal function, anemia, TSH, and MMSE score. The prevalence of Aβ-positivity with normal cognitive function generally increases with age. When considering educational level, a proxy of cognitive reserve, Aβ accumulation is more common in highly educated individuals than in less educated individuals [24]. There is growing evidence linking AD pathology to cerebrovascular and cardiovascular disease, with common risk factors including hypertension in middle age, obesity in middle age, diabetes, hypercholesterolemia, and smoking [25]. Furthermore, the two-hit vascular hypothesis of AD proposes that aging and vascular risk factors lead to dysregulation of the neurovascular unit and are associated with impaired Aβ clearance and increased Aβ production [26, 27]. Although the relationship between cardiovascular disease and AD pathogenesis remains unclear, several studies of autopsy cases suggest that coronary artery disease is associated with intracerebral Aβ accumulation [28]. Regarding the association of liver and renal function with AD, the liver and kidney physiologically remove Aβ from the blood and regulate Aβ levels in the brain; an age-related decline in hepatic Aβ clearance may be involved in the development of AD [29–31]; and eGFR is positively correlated with Aβ42 and Aβ40 and negatively correlated with total-tau levels in CSF [32–34]. An association between thyroid hormones and brain Aβ load has been reported both in vitro and in vivo [35]. In particular, triiodothyronine negatively regulates gene expression of amyloid precursor protein [36]. There are few reports on the association of AD with anemia-related characteristics such as MCV and hemoglobin. MCV has been suggested as a factor associated with cognitive function [37, 38] and previous reports have shown that AD causes changes in erythrocyte morphology. The mechanisms for this include relatively low folate and vitamin B12 concentrations in patients with AD [74], binding of Aβ to erythrocytes, which increases the permeability of the cell membrane and causes fluid accumulation [75–77], changes in the levels of protein components of the cytoskeleton and those linked to the erythrocyte membrane, and changes in the phospholipid composition of the erythrocyte membrane [78, 79]. Low blood hemoglobin (anemia) in older people is associated with cognitive decline via reduced cerebral metabolism, but this appears to be unrelated to aspects of AD-specific protein pathology or cerebrovascular disease as reflected in PET and magnetic resonance imaging measurements [39]. The inclusion of MCV and hemoglobin levels is a novel aspect of our models.

### Limitations

This study had several limitations that should be noted. First, because this was a retrospective observational study, the models were cross-validated internally and have not been validated using external data. Additional studies using external data are needed to verify the classification performance of our models. Second, the model developed in this study is based on two datasets, but relies on data from a limited number of geographical locations and has not been validated using an independent dataset, so external validity and generalizability cannot be guaranteed. Third, because the study models were analyzed in a population that included both MCI and normal cognition, the performances of our developed models are not guaranteed outside of this population. It would be of future interest to investigate individuals with normal cognition with a focus on early detection of Aβ accumulation. Furthermore, the Aβ-positive rate in this study was 39% (101/262), which was lower than existing reports of Aβ-positive rates [80]. Fourth, the lack of data for T3 and T4 levels limits our ability to interpret the association between TSH and amyloid levels. Finally, the model presented in this study showed a moderate performance, so future studies are needed to identify other easily measurable variables (e.g., wearable sensor data [10]) that can be combined with it to further improve performance.

## Conclusions

This study developed machine learning models that are useful for screening PET Aβ-positivity in people with MCI and preclinical AD, providing some novel and interesting insights into the utility of plasma biomarkers in classifying brain amyloid accumulation. Participant demographic characteristics and blood test data accessible to primary care physicians were successfully used to develop a machine learning model to classify PET Aβ-positivity, with a performance of AUC = 0.70. The model using this data combination was more accurate than a model based on basic patient demographic characteristics alone (age, sex, BMI, and years of education) and as accurate as a model based on basic patient demographic characteristics plus MMSE score. Furthermore, adding ApoE4, MMSE score, or both to the blood data slightly improved the classification performance. Unlike other screening models that require specialized tests, we anticipate that our model will be useful for Aβ screening by primary care physicians during routine medical practice because it is easy to use and classifies amyloid PET positivity using routine blood test, interview, and laboratory data. The use of this model as a screening tool is expected to reduce unnecessary PET scans and CSF testing and enable earlier detection and treatment of AD.

## Supplementary Information

Additional file 1. Supplementary Materials Table S1, Table S2, Table S3, and Figure S1.

## Data Availability

The data used in this study cannot be shared publicly owing to ethical restrictions. The consent form signed by the participants states that their data are provided in an anonymized form only to research institutions. The raw data utilized in this study contain sensitive and personally identifiable information that could negatively affect the privacy of the study participants. However, the data supporting the results of this study are available upon ethical approval by the local ethics committee of Oita University Hospital. The ethics committee of Oita University Hospital can be contacted to obtain these data (rinrikenkyu@oita-u.ac.jp).

## Abbreviations

γ-GTP: gamma-glutamyl transferase
Aβ: amyloid β
AA: amino acid
AD: Alzheimer’s disease
ALP: alkaline phosphatase
ALT: alanine transaminase
ApoE4: apolipoprotein E4
AST: aspartate aminotransferase
AUC: area under the curve
BMI: body mass index
CDR: clinical dementia rating
CSF: cerebrospinal fluid
eGFR: estimated glomerular filtration rate
KNN: K-nearest neighbors
MCI: mild cognitive impairment
MCV: mean corpuscular volume
MMSE: Mini Mental State Examination
NC: normal cognitive function
NPV: negative predictive value
PET: positron emission tomography
PiB: ^11^C-Pittsburgh Compound-B
PPV: positive predictive value
ROC: receiver operating characteristic
SD: standard deviation
SHAP: Shapley Additive exPlanations
SMD: standardized mean difference
SUVR: standardized uptake value ratio
SVM: Support Vector Machine
TSH: thyroid-stimulating hormone
WBC: white blood cell
