# Supplementary material for: Machine learning models for dementia screening to classify brain amyloid positivity on positron emission tomography using blood markers and demographic characteristics: a retrospective observational study
Source: Alzheimers Res Ther. 2025 Jan 21;17:25. doi: 10.1186/s13195-024-01650-1 (PMC11748352; doi:10.1186/s13195-024-01650-1)
Supplement: Supplementary file 1 — Additional file 1. Supplementary Materials Table S1, Table S2, Table S3, and Figure S1. [file 13195_2024_1650_MOESM1_ESM.pdf]

Additional file 1. Supplementary Materials

(A)

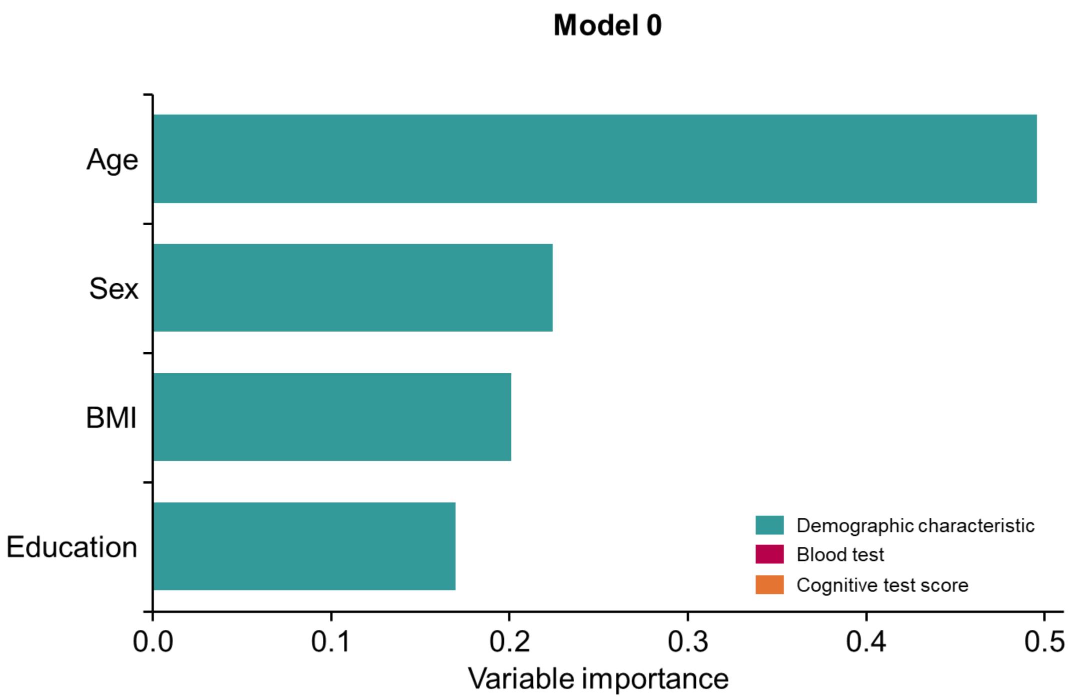

(B)

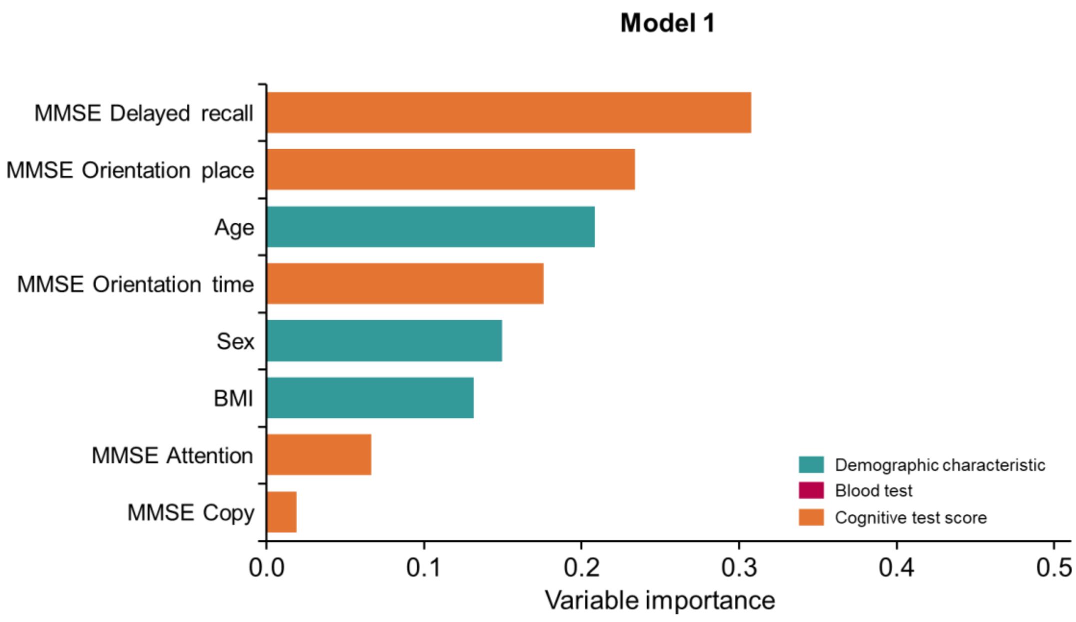

(C)

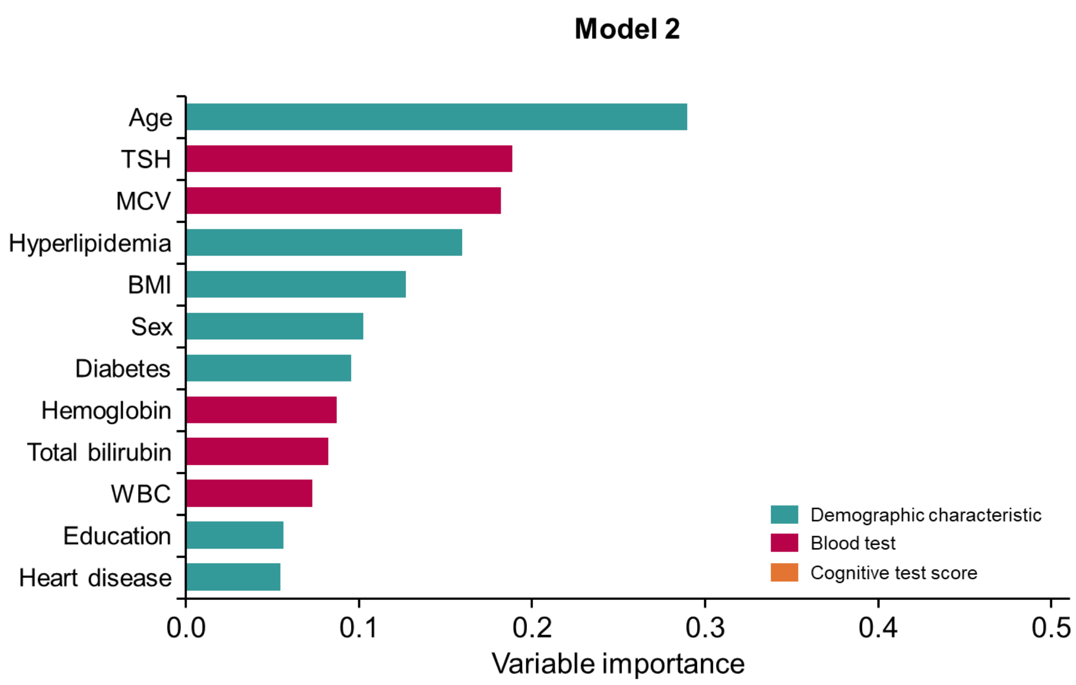

(D)

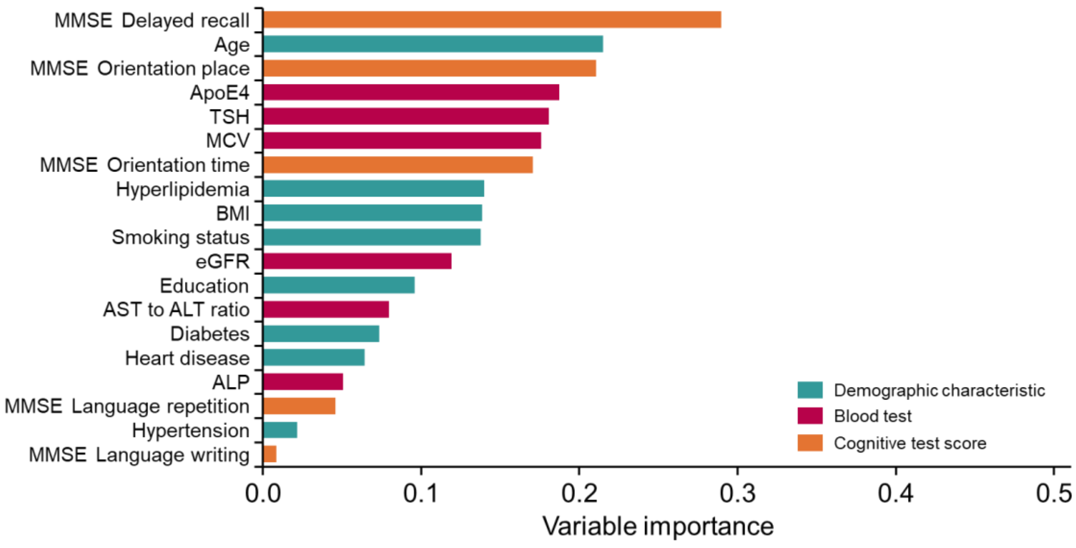

Figure S1. Variable importance in the amyloid β positivity classification models using L2-regularized logistic regression

Model 0: demographic characteristics (age, sex, BMI, years of education)

Model 1: Model 0 plus all MMSE subscores

Model 2: Model 0 plus blood test results (excluding ApoE4 phenotype) and the other demographic characteristics (medical history, current alcohol consumption, smoking status)

Model 4: Model 3 plus ApoE4 phenotype

ALP alkaline phosphatase, *ALT* alanine transaminase, *ApoE4* apolipoprotein E4, *AST* aspartate aminotransferase, *BMI* body mass index, *eGFR* estimated glomerular filtration rate, *MCV* mean corpuscular volume, *MMSE* Mini Mental State Examination, *TSH* thyroid-stimulating hormone, *WBC* white blood cell count

Table S1. Background characteristics of study participants after imputing missing data

	Total<br>*N* = 262[a]	Amyloid β positive<br>*n* = 101	Amyloid β negative<br>*n* = 161	SMD
**Demographic, lifestyle, and clinical characteristics**				
Current alcohol consumption, *n* (%)	94 (35.9)	31 (30.7)	63 (39.1)	0.18
Smoking status[b], *n* (%)	97 (37.0)	29 (28.7)	68 (42.2)	0.29
**Blood test results**				
ApoE4 positive, *n* (%)	50 (19.1)	34 (33.7)	16 (9.9)	0.60
**Cognitive test scores**				
MMSE, mean (SD)				
Orientation time	4.5 (0.8)	4.3 (1.0)	4.7 (0.6)	0.39
Orientation place	4.7 (0.6)	4.5 (0.7)	4.8 (0.5)	0.46
Registration	3.0 (0.1)	3.0 (0.1)	3.0 (0.1)	0.02
Attention	3.4 (1.6)	3.5 (1.5)	3.4 (1.6)	0.09
Delayed recall	2.0 (1.0)	1.8 (1.0)	2.2 (1.0)	0.46
Language naming	2.0 (0.0)	2.0 (0.0)	2.0 (0.0)	<0.01

Language repetition	0.9 (0.2)	0.9 (0.3)	1.0 (0.2)	0.11
Language command	2.9 (0.3)	2.9 (0.4)	2.9 (0.3)	<0.01
Language read and obey	1.0 (0.1)	1.0 (0.1)	1.0 (0.1)	0.07
Language writing	0.9 (0.3)	0.9 (0.3)	0.9 (0.3)	0.07
Copy	1.0 (0.2)	0.9 (0.3)	1.0 (0.2)	0.14

[a]262 records from a total of 260 unique participants

[b]Past or present

ApoE4 apolipoprotein E4, *MMSE* Mini Mental State Examination, *SD* standard deviation, *SMD* standardized mean difference

Table S2. Performance of the amyloid β positivity classification models using other algorithms

	ROC AUC	Sensitivity	Specificity	PPV	NPV	Accuracy
**Model 0[a]**						
Kernel SVM	0.65 (0.02)	0.64 (0.06)	0.63 (0.05)	0.52 (0.02)	0.73 (0.02)	0.63 (0.02)
Elastic Net	0.67 (0.01)	0.64 (0.04)	0.63 (0.04)	0.52 (0.02)	0.74 (0.01)	0.63 (0.01)
**Model 1[b]**						
Kernel SVM	0.70 (0.02)	0.66 (0.07)	0.64 (0.04)	0.54 (0.02)	0.75 (0.03)	0.65 (0.02)
Elastic Net	0.70 (0.01)	0.68 (0.04)	0.62 (0.03)	0.53 (0.01)	0.76 (0.01)	0.64 (0.01)
**Model 2[c]**						
Kernel SVM	0.72 (0.01)	0.68 (0.05)	0.64 (0.04)	0.54 (0.02)	0.76 (0.02)	0.65 (0.02)
Elastic Net	0.70 (0.01)	0.58 (0.04)	0.67 (0.04)	0.54 (0.02)	0.73 (0.01)	0.64 (0.02)
**Model 3[d]**						
Kernel SVM	0.71 (0.02)	0.65 (0.05)	0.64 (0.04)	0.53 (0.02)	0.75 (0.02)	0.64 (0.02)
Elastic Net	0.73 (0.01)	0.62 (0.03)	0.69 (0.03)	0.57 (0.02)	0.75 (0.01)	0.66 (0.01)
**Model 4[e]**						
Kernel SVM	0.75 (0.01)	0.64 (0.03)	0.70 (0.04)	0.59 (0.03)	0.76 (0.01)	0.68 (0.02)
Elastic Net	0.76 (0.01)	0.64 (0.03)	0.75 (0.03)	0.63 (0.03)	0.77 (0.01)	0.71 (0.01)

Data are mean (standard deviation).

[a]Model 0: demographic characteristics (age, sex, body mass index, years of education)

[b]Model 1: Model 0 plus all MMSE subscores

[c]Model 2: Model 0 plus blood test results (excluding ApoE4 phenotype) and the other demographic characteristics (medical history, current alcohol consumption, smoking status)

[d]Model 3: Model 2 plus all MMSE subscores

[e]Model 4: Model 3 plus ApoE4 phenotype

ApoE4 apolipoprotein E4, *MMSE* Mini Mental State Examination*, NPV* negative predictive value, *PPV* positive predictive value, *ROC AUC* receiver operating characteristic area under the curve, *SVM* Support Vector Machine

Table S3. Performance of the amyloid β positivity classification models using the K-nearest neighbors method for imputing missing data

	ROC AUC	Sensitivity	Specificity	PPV	NPV	Accuracy
**Model 0**[a]						
Kernel SVM	0.65 (0.02)	0.63 (0.07)	0.61 (0.06)	0.50 (0.03)	0.73 (0.03)	0.62 (0.02)
Elastic Net	0.67 (0.01)	0.65 (0.04)	0.62 (0.03)	0.52 (0.02)	0.74 (0.01)	0.64 (0.01)
Logistic Regression	0.67 (0.01)	0.66 (0.03)	0.62 (0.03)	0.52 (0.02)	0.74 (0.01)	0.63 (0.01)
**Model 1**[b]						
Kernel SVM	0.70 (0.02)	0.65 (0.06)	0.64 (0.03)	0.54 (0.03)	0.74 (0.03)	0.64 (0.02)
Elastic Net	0.71 (0.01)	0.67 (0.03)	0.61 (0.02)	0.52 (0.01)	0.75 (0.01)	0.63 (0.01)
Logistic Regression	0.71 (0.01)	0.67 (0.02)	0.62 (0.02)	0.53 (0.01)	0.75 (0.01)	0.63 (0.01)
**Model 2**[c]						
Kernel SVM	0.68 (0.02)	0.57 (0.06)	0.66 (0.05)	0.52 (0.03)	0.71 (0.02)	0.63 (0.02)
Elastic Net	0.70 (0.01)	0.58 (0.04)	0.70 (0.04)	0.57 (0.03)	0.73 (0.01)	0.66 (0.02)

Logistic Regression	0.70 (0.01)	0.54 (0.04)	0.73 (0.03)	0.57 (0.03)	0.72 (0.01)	0.66 (0.01)
**Model 3[d]**						
Kernel SVM	0.71 (0.02)	0.65 (0.04)	0.66 (0.04)	0.55 (0.02)	0.75 (0.02)	0.66 (0.02)
Elastic Net	0.74 (0.01)	0.64 (0.03)	0.68 (0.03)	0.56 (0.02)	0.76 (0.01)	0.66 (0.01)
Logistic Regression	0.73 (0.01)	0.63 (0.03)	0.70 (0.03)	0.57 (0.02)	0.75 (0.02)	0.67 (0.02)
**Model 4[e]**						
Kernel SVM	0.76 (0.01)	0.62 (0.04)	0.75 (0.05)	0.63 (0.04)	0.76 (0.01)	0.70 (0.02)
Elastic Net	0.75 (0.01)	0.62 (0.03)	0.78 (0.03)	0.65 (0.03)	0.77 (0.01)	0.72 (0.02)
Logistic Regression	0.75 (0.01)	0.55 (0.03)	0.83 (0.02)	0.68 (0.03)	0.75 (0.01)	0.72 (0.01)

Data are mean (standard deviation).

[a]Model 0: demographic characteristics (age, sex, body mass index, years of education)

[b]Model 1: Model 0 plus all MMSE subscores

[c]Model 2: Model 0 plus blood test results (excluding ApoE4 phenotype) and the other demographic characteristics (medical history, current alcohol consumption, smoking status)

[d]Model 3: Model 2 plus all MMSE subscores

[e]Model 4: Model 3 plus ApoE4 phenotype

ApoE4 apolipoprotein E4, *MMSE* Mini Mental State Examination*, NPV* negative predictive value, *PPV* positive predictive value, *ROC AUC* receiver operating characteristic area under the curve, *SVM* Support Vector Machine
